# COVID-19 Vaccine Hesitancy: Umbrella Review of Systematic Reviews and Meta-Analysis

**DOI:** 10.2196/54769

**Published:** 2024-04-30

**Authors:** Tahani Al Rahbeni, Prakasini Satapathy, Ramaiah Itumalla, Roy Rillera Marzo, Khalid A L Mugheed, Mahalaqua Nazli Khatib, Shilpa Gaidhane, Quazi Syed Zahiruddin, Ali A Rabaan, Hayam A Alrasheed, Maha F Al-Subaie, Nawal A Al Kaabil, Mohammed Alissa, Amani Ahmed A L Ibrahim, Hussain Abdulkhaliq Alsaif, Israa Habeeb Naser, Sarvesh Rustagi, Neelima Kukreti, Arkadiusz Dziedzic

**Affiliations:** 1 Molecular Toxicology and Genetics Riyadh Elm University Riyadh Saudi Arabia; 2 Center for Global Health Research Saveetha Medical College and Hospital Saveetha Institute of Medical and Technical Sciences, Saveetha University Chennai India; 3 School of Management The Apollo University Chittoor India; 4 Faculty of Humanities and Health Sciences Curtin University Miri Sarawak Malaysia; 5 Division of Evidence Synthesis Global Consortium of Public Health and Research Datta Meghe Institute of Higher Education Wardha India; 6 One Health Centre (COHERD) Jawaharlal Nehru Medical College Datta Meghe Institute of Higher Education Wardha India; 7 South Asia Infant Feeding Research Network Division of Evidence Synthesis School of Epidemiology and Public Health and Research, Jawaharlal Nehru Medical College, Datta Meghe Institute of Higher Education and Research Wardha India; 8 Molecular Diagnostic Laboratory Johns Hopkins Aramco Healthcare Dhahran Saudi Arabia; 9 College of Medicine Alfaisal University Riyadh Saudi Arabia; 10 Department of Public Health and Nutrition The University of Haripur Haripur Pakistan; 11 Department of Pharmacy Practice College of Pharmacy Princess Nourah bint Abdulrahman University Riyadh Saudi Arabia; 12 Research Center Dr Sulaiman Alhabib Medical Group Riyadh Saudi Arabia; 13 College of Medicine and Health Science Khalifa University Abu Dhabi United Arab Emirates; 14 Sheikh Khalifa Medical City Abu Dhabi Health Services Company (SEHA) Abu Dhabi United Arab Emirates; 15 Department of Medical Laboratory College of Applied Medical Sciences Prince Sattam bin Abdulaziz University Al-Kharj Saudi Arabia; 16 Deparment of Pharmacy Jubail General Hospital Jubail Saudi Arabia; 17 Department of Medicine Batterjee Medical College Jeddah Saudi Arabia; 18 Medical Laboratories Techniques Department AL-Mustaqbal University Babil Iraq; 19 School of Applied and Life Sciences Uttaranchal University Dehradun India; 20 School of Pharmacy Graphic Era Hill University Dehradun India; 21 Department of Conservative Dentistry with Endodontics Medical University of Silesia Katowice Poland

**Keywords:** COVID-19, vaccine acceptance, vaccine hesitancy, umbrella review, systematic review, meta-analysis, vaccine, hesitancy, global perceptions, perception, random effect model, synthesis, healthcare workers, patients, patient, chronic disease, pregnant women, parents, child, children

## Abstract

**Background:**

The unprecedented emergence of the COVID-19 pandemic necessitated the development and global distribution of vaccines, making the understanding of global vaccine acceptance and hesitancy crucial to overcoming barriers to vaccination and achieving widespread immunization.

**Objective:**

This umbrella review synthesizes findings from systematic reviews and meta-analyses to provide insights into global perceptions on COVID-19 vaccine acceptance and hesitancy across diverse populations and regions.

**Methods:**

We conducted a literature search across major databases to identify systematic reviews and meta-analysis that reported COVID-19 vaccine acceptance and hesitancy. The AMSTAR-2 (A Measurement Tool to Assess Systematic Reviews) criteria were used to assess the methodological quality of included systematic reviews. Meta-analysis was performed using STATA 17 with a random effect model. The data synthesis is presented in a table format and via a narrative.

**Results:**

Our inclusion criteria were met by 78 meta-analyses published between 2021 and 2023. Our analysis revealed a moderate vaccine acceptance rate of 63% (95% CI 0.60%-0.67%) in the general population, with significant heterogeneity (*I*^2^ = 97.59%). Higher acceptance rates were observed among health care workers and individuals with chronic diseases, at 64% (95% CI 0.57%-0.71%) and 69% (95% CI 0.61%-0.76%), respectively. However, lower acceptance was noted among pregnant women, at 48% (95% CI 0.42%-0.53%), and parents consenting for their children, at 61.29% (95% CI 0.56%-0.67%). The pooled vaccine hesitancy rate was 32% (95% CI 0.25%-0.39%) in the general population. The quality assessment revealed 19 high-quality, 38 moderate-quality, 15 low-quality, and 6 critically low-quality meta-analyses.

**Conclusions:**

This review revealed the presence of vaccine hesitancy globally, emphasizing the necessity for population-specific, culturally sensitive interventions and clear, credible information dissemination to foster vaccine acceptance. The observed disparities accentuate the need for continuous research to understand evolving vaccine perceptions and to address the unique concerns and needs of diverse populations, thereby aiding in the formulation of effective and inclusive vaccination strategies.

**Trial Registration:**

PROSPERO CRD42023468363; https://tinyurl.com/2p9kv9cr

## Introduction

The global health landscape has been profoundly altered by the emergence of COVID-19, triggered by SARS-CoV-2. First identified in Wuhan, China, in December 2019, this virulent pathogen swiftly traversed continents, leading the World Health Organization (WHO) to categorize the situation as both a pandemic and public health emergency of international concern. The repercussions of this pandemic have been multifaceted, with a staggering death toll and profound impact on socioeconomic structures worldwide [1]. In the face of this unprecedented challenge, the global community recognized the pressing need for effective countermeasures. Although therapeutic interventions were explored, the primary focus shifted to preventive strategies, with vaccines against COVID-19 emerging as the most promising solution [2]. The efficacy of this approach, however, is contingent not just on the scientific success of vaccine development but equally on the global populace's acceptance of these vaccines [3].

By the midpoint of 2022, the scientific community had successfully developed, trialed, and secured emergency use authorization for several vaccines [4]. However, the distribution of these vaccines unveiled pronounced disparities [5]. Higher-income nations, with their robust health care infrastructures and financial resources, rapidly initiated vaccination drives. In stark contrast, many resource-limited countries faced challenges ranging from limited vaccine access to infrastructural constraints [6]. A more insidious challenge that emerged globally, irrespective of a country's economic status, was vaccine hesitancy. Rooted in a complex interplay of factors, including safety apprehensions, distrust toward health advisories, cultural nuances, and the deluge of misinformation, vaccine hesitancy has been observed across diverse geographies, from Africa and Europe to North America [3].

Empirical studies conducted across various regions have painted a mixed picture of vaccine acceptance [3,7]. Although certain demographics exhibited a commendable eagerness to embrace vaccination, others displayed pronounced skepticism [3,8]. These disparities in vaccine acceptance, if unchecked, have the potential to impede global strides toward achieving herd immunity, a critical milestone in the fight against the pandemic. Recognizing the pivotal role of vaccine acceptance in the trajectory of the pandemic, it becomes imperative to understand the nuances of global vaccine perceptions. Numerous systematic reviews and meta-analyses have been published, shedding light on factors that are driving vaccine hesitancy and acceptance [8-17].

In this context, our umbrella review sought to collate and synthesize findings from these diverse studies, aiming to present a holistic understanding of global COVID-19 vaccine acceptance rates and hesitancy rates. This approach offers a comprehensive overview of existing evidence, highlighting consistencies and discrepancies across studies. By assessing the quality and breadth of current research, umbrella reviews identify knowledge gaps and inform evidence-based decision-making. They serve as a valuable tool for policymakers, clinicians, and researchers, providing a holistic understanding of a topic without the need for sifting through numerous individual studies. Through this study, we aspired to provide valuable insights that can steer future vaccination strategies, ensuring they are both effective and inclusive. Our umbrella review aimed to collate and synthesize findings from these diverse studies to present a comprehensive understanding of global COVID-19 vaccine acceptance and hesitancy rates.

## Methods

The method for conducting this umbrella review was based on the framework set forth by the Joanna Briggs Institute [18]. This study adhered to the PRISMA (Preferred Reporting Items for Systematic Reviews and Meta-Analyses) guideline [19] (Multimedia Appendix 1). This study was registered in PROSPERO.

### Inclusion Criteria

We specifically targeted meta-analyses of epidemiological studies that investigated either the acceptance/willingness or hesitancy toward the COVID-19 vaccine. Our scope was global, encompassing studies from all geographical locations without any specific focus on a particular population. This inclusivity ensured that our review captured a diverse range of perspectives and settings. However, to maintain the rigor and specificity of our review, we excluded certain types of publications. Specifically, narrative or systematic reviews that did not include a meta-analysis, conference abstracts, and letters to the editors were not considered. In essence, our inclusion criteria were centered on meta-analyses of prospective, retrospective, or cross-sectional studies that evaluated rates of vaccine acceptance or hesitancy (Table S1 in Multimedia Appendix 2).

### Literature Search

We conducted a comprehensive literature search across 4 major databases: PubMed, Scopus, Embase, and the Cochrane Database of Systematic Reviews. The search spanned from the inception of each database until August 20, 2023. To ensure a thorough retrieval of relevant articles, “keyword search” and “textword search” were used, and different search phrases were combined using Boolean and proximity operators. Specifically, we used the terms (“meta-analysis” OR “systematic review”) AND (Acceptance OR willingness OR hesitancy OR intention OR unwillingness) AND (“COVID-19” OR “Sars-cov-2” OR “corona*”). To further enhance the robustness of our search, we also manually screened the reference lists of the identified articles. This step ensured that we did not overlook any pertinent studies that might not have been captured through the database search. For transparency and replicability, the complete search strategy, including all terms and combinations used, is documented in Table S2 in Multimedia Appendix 2. Importantly, we did not impose any filters or restrictions during our search. This means that articles of any type, published in any year, and in any language were considered, ensuring a broad and inclusive search scope.

### Screening

The screening process for this systematic review was conducted by 2 independent authors, structured into 2 sequential steps to ensure unbiased selection and comprehensive coverage of relevant studies. The first step, primary screening, involved scrutinizing the titles and abstracts of identified articles to shortlist those potentially relevant to topic. Subsequently, in the second step, articles that passed the primary screening were subjected to a thorough full-text review. During this stage, the authors carefully evaluated the complete content of each article, focusing on the removal of duplicates and a more detailed assessment of each study's relevance and alignment with the review's scope and objectives. To enhance the precision and efficiency of our screening process, we used the specialized software Nested Knowledge with its AutoLit function, instrumental in streamlining our workflow and improving the accuracy of article selection. In cases of disagreement or uncertainty between the 2 reviewers, a third reviewer with senior expertise was engaged to mediate and provide decisive judgment, ensuring a consensus-based approach to the final selection of studies.

### Data Extraction

During the data extraction process, 2 independent authors systematically reviewed each study that met our inclusion criteria. From each eligible meta-analysis, they gathered a comprehensive set of details. This included the first author's name, year of publication, type of study design, total number of participants, type of population, and the date when the database search was conducted. Additionally, they extracted the pooled acceptance rate for each subgroup and specific number of participants within these subgroups, accompanied by their 95% CIs. Furthermore, they documented the *P* values for pooled effects, the results from the Egger or Beggs test (which measures publication bias), and the *I*^2^ statistics, which offer insights into the heterogeneity of the studies. Any associated *P* value for significance was also recorded. Given the complexity of the data and the importance of accuracy, any discrepancies or disagreements that arose between the 2 primary authors were diligently addressed. They consulted a third, senior reviewer to ensure consistency and precision in the data extraction process.

### Assessment of Methodological Quality

To ensure the rigor and reliability of our review, the methodological quality of each included meta-analysis was meticulously evaluated. This assessment was jointly undertaken by 2 authors using the well-established AMSTAR-2 (A Measurement Tool to Assess Systematic Reviews) tool [20], which is recognized for its robustness in appraising systematic reviews. Based on the criteria set by AMSTAR-2, studies were categorized into 1 of 4 methodological quality grades: high, moderate, low, or critically low. A study was deemed to be of high quality if it exhibited no flaws or only a single minor defect. In contrast, a moderate quality designation was given to studies that presented multiple minor defects. The distinction between minor and major defects was made based on the guidelines provided by the AMSTAR-2 tool. Any disagreements or uncertainties regarding the quality grading were discussed and resolved collaboratively between the 2 authors to ensure a consistent and objective assessment.

### Data Analysis

Data analysis was conducted using STATA version 17. Proportions, along with their 95% CIs, were pooled from all included eligible meta-analyses for each outcome and based on the population [21]. We used a random effects model to compute the combined effect sizes, recognizing the inherent variability among the studies and providing a more conservative estimate of the overall effect. The degree of variability or heterogeneity in outcomes across studies was quantified using the *I*^2^ metric. Values for *I*^2^ can range from 0% to 100%, with higher values indicating greater heterogeneity. We predetermined specific thresholds to assess the statistical significance of the observed heterogeneity. The 95% prediction interval provides a more comprehensive understanding of the range in which the true effect size lies, considering the observed heterogeneity. A *P* value <0.05 was considered statistically significant.

## Results

### Search Results

**Figure 1:**
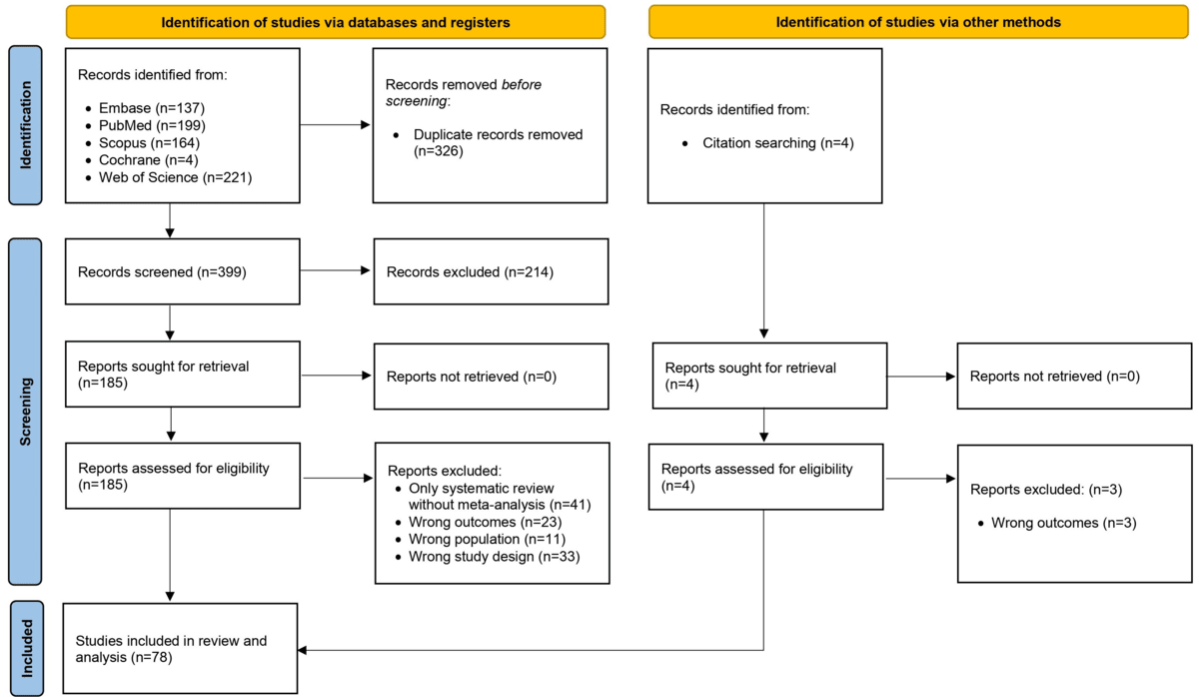
PRISMA (Preferred Reporting Items for Systematic Reviews and Meta-Analyses) flow diagram illustrating the screening and selection process.

We identified a total of 662 articles through the primary search, of which 263 duplicates were eliminated, leaving 399 for title and abstract screening. In the primary screening (title and abstract), 214 articles were excluded, leaving 185 articles for full-text screening (Figure 1). We excluded 108 articles for various reasons, including only systematic reviews without a meta-analysis and the incorrect population, outcomes, or study design. As a result, 77 articles fulfilled the eligibility criteria. Additionally, we conducted a citation search to maintain the rigor of the review and found 4 relevant articles, of which 1 was included. This umbrella review ultimately identified 78 meta-analyses that met the inclusion criteria.

### Characteristics of Meta-Analyses and Quality Assessment

Table S3 in Multimedia Appendix 2 [9-17,22-90] presents an overview of all included meta-analyses published between 2021 and 2023. These studies looked at different groups, such as the general population; health care workers; parents; pregnant women; migrant workers; Black or African communities; Chinese communities; and people with specific health conditions such as cancer, HIV, diabetes, inflammatory bowel disease, and epilepsy. The focus of these meta-analyses was on outcomes such as the rates of vaccine acceptance, hesitance, willingness, uncertainty, and unwillingness, as well as the intention to receive vaccines. Most of the meta-analyses included articles from around the world, while some concentrated on specific countries.

The quality of these meta-analyses was evaluated using the AMSTAR-2 criteria. Among the meta-analyses examined, 19 meta-analyses were rated as being of high quality; 38 meta-analyses received a moderate quality rating; 15 meta-analyses received a low quality rating, which points to potential limitations in their methodology; and 6 meta-analyses were classified as having critically low quality, implying significant concerns about their methods and the reliability of their findings (Table S4 in Multimedia Appendix 2 [9-17,22-90]).

### Vaccine Acceptance and Hesitancy Among Populations

Table 1 provides a summary of vaccine acceptance and hesitancy rates among different populations. Acceptance rates were studied in 58 systematic reviews and meta-analyses, and 12 distinct reviews reported on hesitancy rates (Table 2).

**Table 1:** Summary of vaccine acceptance rates across different populations.

Studies by population			Acceptance rate, % (95% CI)		Heterogeneity (*I*^2^), %			Overall acceptance rate, % (95% CI)	
**General population**						97.59			0.63 (0.60-0.67)
	Wang et al, 2021 [22]	0.74 (0.71-0.77)							
	Alimohamadi et al, 2022 [10]	0.67 (0.62-0.74)							
	Abdelmoneim et al, 2022 [23]	0.81 (0.75-0.85)							
	Nehal et al, 2021 [15]	0.66 (0.6-0.7)							
	Khabour, 2022 [24]	0.39 (0.33-0.46)							
	Sahile et al, 2022 [25]	0.57 (0.47-0.67)							
	Norhayati et al, 2021 [12]	0.61 (0.59-0.64)							
	Wake, 2021 [26]	0.48 (0.39-0.58)							
	Alarcón-Braga et al, 2022 [27]	0.78 (0.74-0.82)							
	Mekonnen and Mengistu, 2022 [28]	0.56 (0.47-0.64)							
	Mengistu et al, 2022 [29]	0.64 (0.6-0.69)							
	Gudayu and Mengistie, 2023 [30]	0.68 (0.67-0.68)							
	Kumar et al, 2023 [16]	0.62 (0.55-0.69)							
	Alemayehu et al, 2022 [31]	0.60 (0.52-0.67)							
	Kawuki et al, 2023 [32]	0.58 (0.49-0.67)							
	Wang et al, 2021 [22]	0.67 (0.67-0.68)							
	Belay et al, 2022 [33]	0.51 (0.43-0.58)							
	Robinson et al, 2021 [34]	0.72 (0.66-0.78)							
	Mahmud et al, 2022 [35]	0.62 (0.58-0.66)							
	Azanaw et al, 2022 [36]	0.55 (0.47-0.62)							
	Terry et al, 2022 [37]	0.73 (0.64-0.81)							
	Yenew et al, 2023 [38]	0.67 (0.60-0.74)							
	Kukreti et al, 2022 [39]	0.60 (0.51-0.68)							
	Nnaemeka et al, 2023 [40]	0.52 (0.46-0.57)							
	Akem Dimala et al, 2021 [41]	0.71 (0.66-0.76)							
	Renzi et al, 2022 [42]	0.66 (0.61-0.71)							
	Kazeminia et al, 2022 [43]	0.63 (0.59-0.68)							
	Mose et al, 2022 [44]	0.51 (0.43-0.59)							
	Yanto et al, 2022 [9]	0.71 (0.69-0.74)							
**Chronic disease**						87.50			0.69 (0.61-0.76)
	Wang et al, 2021 [22]	0.85 (0.82-0.88)							
	Yazdani et al, 2022 [45]	0.76 (0.67-0.85)							
	Zhao et al, 2023 [46]	0.65 (0.59-0.72)							
	Lin et al, 2022 [47]	0.58 (0.45-0.75)							
	Meybodi et al, 2022 [48]	0.59 (0.39-0.79)							
	Ejamo et al, 2023 [49]	0.62 (0.56-0.69)							
	Ekpor and Akyirem, 2023 [50]	0.76 (0.66-0.83)							
	Prabani et al, 2022 [51]	0.59 (0.52-0.67)							
**Health care workers**						91.72			0.64 (0.57-0.71)
	Wang et al, 2021 [22]	0.65 (0.55-0.75)							
	Luo et al, 2021 [11]	0.51 (0.41-0.62)							
	Alimohamadi et al, 2022 [10]	0.55 (0.47-0.64)							
	Shui et al, 2022 [52]	0.78 (0.73-0.83)							
	Lin et al, 2022 [85]	0.81 (0.72-0.89)							
	Ackah et al, 2022 [53]	0.46 (0.37-0.54)							
	Moltot et al, 2023 [54]	0.54 (0.42-0.66)							
	Ulbrichtova et al, 2022 [55]	0.71 (0.67-0.75)							
	Politis et al, 2023 [56]	0.64 (0.55-0.72)							
	Wang et al, 2022 [13]	0.66 (0.61-0.67)							
**Pregnant women**						74.20			0.48 (0.42-0.53)
	Sarantaki et al, 2022 [57]	0.53 (0.44-0.61)							
	Nikpour et al, 2022 [58]	0.54 (0.45-0.62)							
	Nassr et al, 2022 [59]	0.47 (0.38-0.57)							
	Halemani et al, 2022 [60]	0.54 (0.46-0.61)							
	Shamshirsaz et al, 2022 [61]	0.47 (0.38-0.57)							
	Galanis et al, 2022 [62]	0.27 (0.18-0.37)							
	Bhattacharya et al, 2022 [63]	0.49 (0.42-0.56)							
	Worede et al, 2023 [14]	0.42 (0.28-0.56)							
	Azami et al, 2022 [64]	0.53 (0.47-0.59)							
**Parents regarding vaccinating their children**						50.29			0.61 (0.56-0.67)
	Wang et al, 2022 [65]	0.58 (0.28-0.98)							
	Galanis et al, 2022 [66]	0.6 (0.517-0.68)							
	Chen et al, 2022 [67]	0.61 (0.53-0.68)							
	Ma et al, 2022 [68]	0.7 (0.62-0.78)							
	Alimoradi et al, 2023 [69]	0.57 (0.52-0.62)							
**Migrants and refugees**						74.04			0.69 (0.56-0.82)
	Alimoradi et al, 2023 [70]	0.7 (0.62-0.77)							
	Hajissa et al, 2023 [71]	0.56 (0.449-0.685)							
**Chinese community residents**						N/A^a^			0.80 (0.72-0.88)
	Xu and Zhu, 2022 [72]	0.8 (0.71-0.87)							

^a^N/A: not applicable.

**Table 2:** Summary of vaccine hesitancy rates across different populations.

Studies by population		Hesitancy rate, % (95% CI)	Heterogeneity (*I*^2^), %		Overall hesitancy rate, % (95% CI)	
**General population**				73.90		0.32 (0.25-0.39)
	Patwary et al, 2022 [73]	0.382 (0.272-0.497)				
	Islam et al, 2023 [17]	0.265 (0.22-0.31)				
	Kawuki et al, 2023 [32]	0.29 (0.18-0.43)				
	Fajar et al, 2022 [74]	0.25 (0.19-0.32)				
	Cénat et al, 2022 [75]	0.423 (0.337-0.51)				
**Older adults**				N/A^a^		0.27 (0.16-0.39)
	Veronese et al, 2021 [76]	0.27 (0.15-0.38)				
**Black/African American people**				N/A		0.35 (0.25-0.44)
	Ripon et al, 2022 [77]	0.35 (0.26-0.45)				
**People with diabetes**				N/A		0.27 (0.14-0.40)
	Bianchi et al, 2023 [84]	0.27 (0.156-0.419)				
**Health care students**				N/A		0.26 (0.18-0.33)
	Patwary et al, 2022 [79]	0.258 (0.185-0.338)				
**Pregnant and breastfeeding women**				N/A		0.48 (0.43-0.53)
	Bianchi et al, 2022 [80]	0.484 (0.434-0.534)				
**Parents regarding vaccinating their children**				95.79		0..39 (0.07-0.70)
	Bianchi et al, 2023 [81]	0.55 (0.43-0.66)				
	Galanis et al, 2022 [66]	0.229 (0.173-0.29)				
**Migrants**				N/A		0.31 (0.21-0.41)
	Hajissa et al, 2023 [71]	0.31 (0.215-0.42)				
**Health care workers**				97.30		0.29 (0.18-0.33)
	Bianchi et al, 2022 [78]	0.13 (0.069-0.209)				
	Kigongo et al, 2023 [82]	0.46 (0.38-0.54)				

^a^N/A: not applicable.

### Vaccine Acceptance

**Figure 2:**
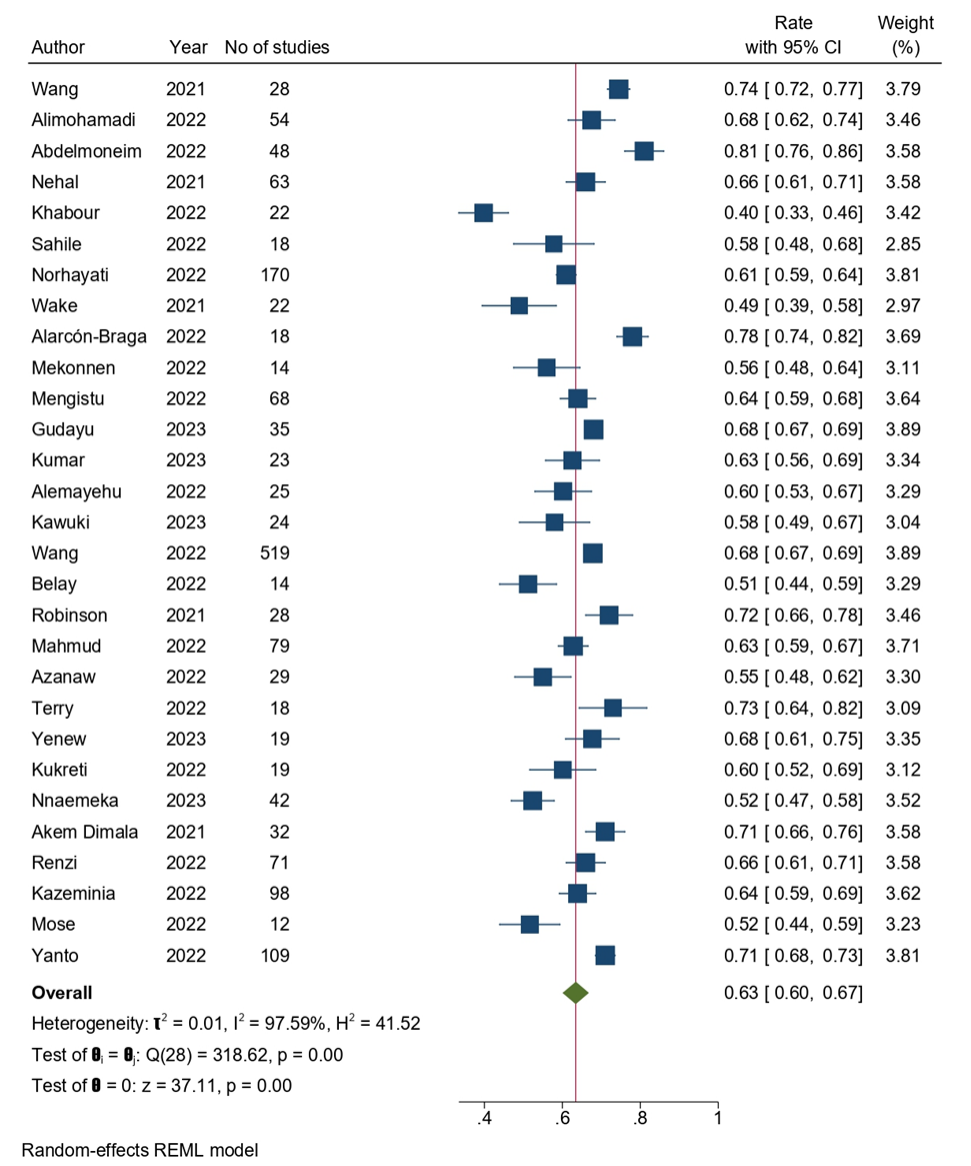
Forest plot depicting the pooled acceptance rates for COVID-19 vaccines in the general population. REML: restricted maximum likelihood.

We synthesized findings from 29 systematic reviews to assess the vaccine acceptance rate in the general population. The pooled acceptance rates ranged from 51% to 81%. Our meta-analysis revealed a consolidated vaccine acceptance rate of 63% (95% CI 0.60%-0.67%). Notably, a high level of heterogeneity was observed, with an *I*^2^ of 97.59% (Figure 2). The 8 systematic reviews focused on individuals with chronic diseases reported a pooled acceptance rate of 69% (95% CI 0.61%-0.76%) and an *I*^2^ of 87.5% (Multimedia Appendix 3). Health care workers were the focus of 10 systematic reviews and meta-analyses, indicating a 64% acceptance rate (95% CI 0.57%-0.71%) and a heterogeneity *I*^2^ of 91.72% (Multimedia Appendix 4). Vaccine acceptance was comparatively lower among pregnant women, as depicted by 9 systematic reviews, showing a rate of 48% (95% CI 0.42%-0.53%) and an *I*^2^ of 74.2% **(**Multimedia Appendix 5). Similarly, 5 systematic reviews presented a 61.29% acceptance rate (95% CI 0.56%-0.67%) with 50% heterogeneity for parents consenting for their children (Multimedia Appendix 6). Vaccine acceptance among migrants and refugees was investigated by 2 reviews, showing a prevalence of 69% (95% CI 0.56%-0.82%) with an *I*^2^ of 74%, and 1 review focused on the Chinese community, reporting an 80% acceptance rate (95% CI 0.72%-0.88%; Multimedia Appendix 7).

### Vaccine Hesitancy

**Figure 3:**
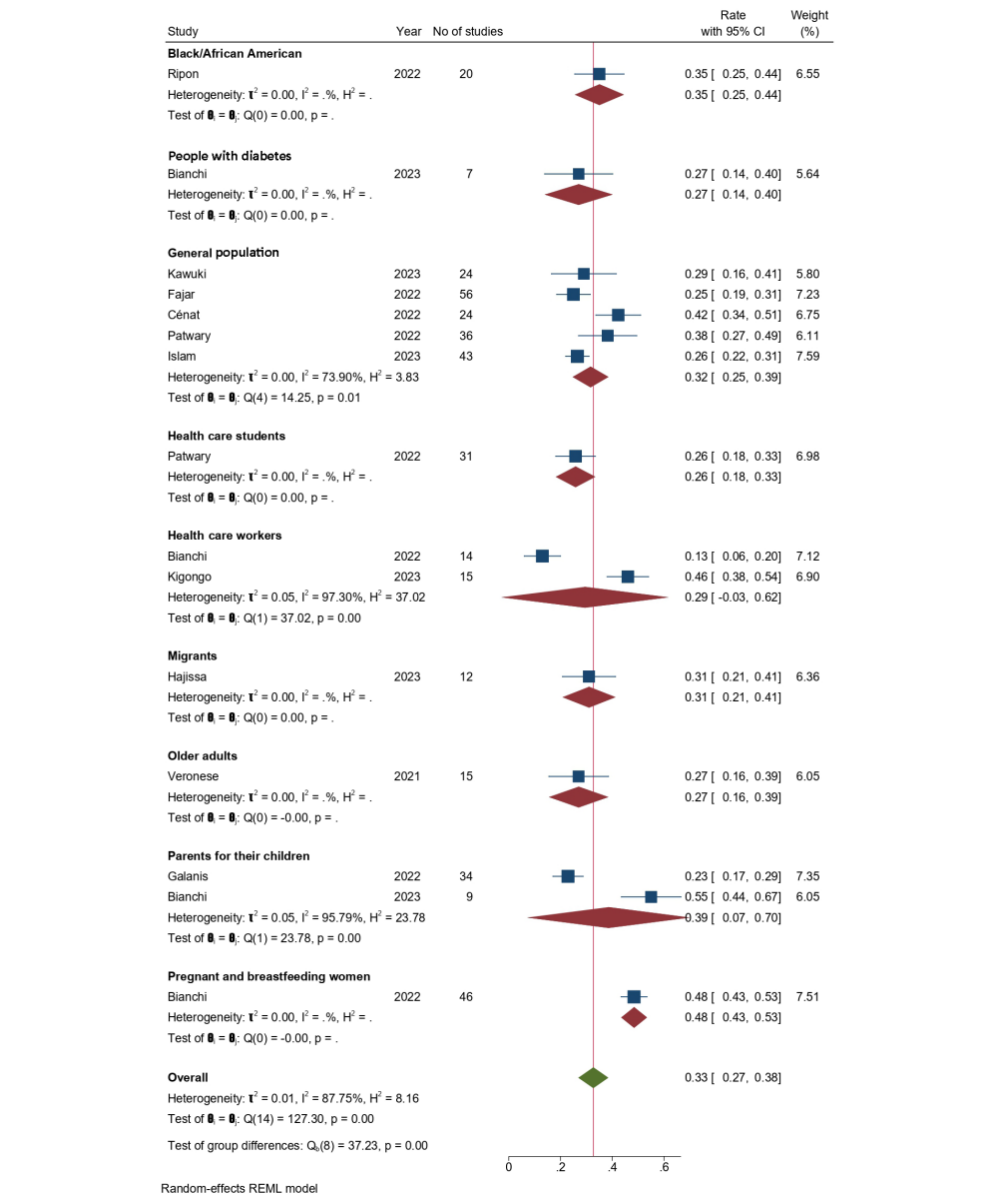
Forest plot showing COVID-19 vaccine hesitancy rates for different populations. REML: restricted maximum likelihood.

Figure 3 shows the forest plot of COVID-19 vaccine hesitancy for different populations. Vaccine hesitancy in the general population was reported by 5 systematic reviews, with the observed hesitancy varying between 25% and 42%. Our meta-analysis showed a pooled vaccine hesitancy rate of 32% (95% CI 0.25%-0.39%), with a high level of heterogeneity (*I*^2^=73.90%). In older adults, 1 review reported a hesitancy rate of 27% (95% CI 0.15%-0.38%). For Black people or African Americans, vaccine hesitancy was 35% (95% CI 0.26%-0.45%) in another review. Pregnant or breastfeeding women exhibited a higher hesitancy rate of 48.4% (95% CI 0.43%-0.53%), as reported by another systematic review. Results on the hesitancy rates among parents considering vaccinating their children were provided by 2 reviews, revealing a pooled hesitancy rate of 39% (95% CI 0.07%-0.70%), accompanied by a high level of heterogeneity (*I*^2^=95.7%). Migrant workers exhibited a hesitancy rate of 31% (95% CI 0.21%-0.41%) according to 1 study. Last, health care workers showed a rate of 29% (95% CI 0.18%-0.33%), as concluded from 2 systematic reviews.

## Discussion

Our umbrella review synthesized findings from numerous systematic reviews and meta-analyses, providing insights into global vaccine acceptance and hesitancy rates across diverse populations and geographies. The consolidated acceptance rate of 63% in the general population indicates a moderate level of willingness to receive the vaccine.

The emergence of the COVID-19 pandemic necessitated the prompt development and distribution of vaccines to curb the spread of the virus and mitigate its adverse impacts on global health, economies, and societies [91]. As of the midpoint of 2021, several vaccines had received emergency use authorization, signifying a milestone in the fight against the pandemic. However, the realization of the potential of these vaccines is significantly influenced by the global population's acceptance and willingness to get vaccinated [92,93]. However, the significant heterogeneity observed in this and other populations studied underscores the diverse and complex landscape of vaccine perceptions globally [53]. The vaccine acceptance rates among health care workers and individuals with chronic diseases were relatively higher, possibly reflecting a better understanding of the disease's risk and the vaccine's benefits by these populations [94,95]. However, the observed heterogeneity suggests diverse opinions and possibly varied information dissemination within these groups. The disparities in vaccine acceptance and hesitancy across populations are emblematic of the intricate tapestry of perceptions, beliefs, and information access that characterize the global populace. For instance, the lower acceptance rates observed among pregnant women and parents consenting for their children are likely influenced by concerns about vaccine safety in these vulnerable groups, emphasizing the need for targeted communication strategies addressing these concerns [96].

The pronounced disparities in vaccine acceptance across different populations highlight the urgent need for tailored, population-specific intervention strategies [97]. A one-size-fits-all approach may not address the unique concerns, misconceptions, and information needs of different demographic groups. For example, pregnant women exhibited lower acceptance and higher hesitancy rates, possibly due to concerns regarding the vaccine's impact on pregnancy and the fetus [98,99]. Addressing such specific concerns through targeted awareness campaigns and counseling can enhance vaccine acceptance in this group. Similarly, the lower acceptance rates in parents consenting for their children necessitate interventions addressing parental concerns about vaccine safety and efficacy in children [100]. Engaging pediatricians and child health care providers in vaccine advocacy can potentially alleviate parental apprehensions and foster trust. The high heterogeneity observed across studies denotes the existence of multiple influencing factors, including cultural, socioeconomic, educational, and individual beliefs, which vary extensively within and across populations [101,102]. The variations may also reflect the differences in study designs, populations, and time frames, emphasizing the need for standardization in future research to facilitate comparability and generalizability [103].

The emergence of vaccine hesitancy as a global phenomenon irrespective of a country's economic status underscores the influential role of information dissemination and public perception in shaping vaccine-related behaviors [104,105]. Misinformation and distrust in health advisories have been pivotal in fostering hesitancy, indicating the need for credible, clear, and consistent communication from health authorities and governments [106-110]. Addressing misinformation necessitates a multifaceted approach involving the collaboration of health care providers, public health officials, social media platforms, and community leaders. The propagation of accurate, comprehensible, and transparent information regarding vaccine development, efficacy, and safety can contribute to counteracting misinformation. Health care workers, with an acceptance rate of 64%, play a crucial role in shaping public perceptions and behaviors regarding vaccination [111]. As trusted sources of health information, health care providers can effectively address concerns, clarify misconceptions, and advocate for the benefits of vaccination [63,68]. Their interactions with patients and communities can significantly influence vaccine acceptance, especially in populations with high hesitancy levels, such as pregnant women and parents. However, the hesitancy rate of 29% among health care workers is concerning, as it can potentially impact their vaccine advocacy efforts. Addressing the concerns and information needs of health care workers is imperative to fostering confidence in vaccines and enhancing their role as vaccine advocates.

The acceptance and hesitancy rates in specific communities, such as Black or African American and Chinese communities, underscore the impact of cultural and community nuances on vaccine perceptions [112,113]. Culturally sensitive approaches, community engagement, and addressing systemic barriers are essential to enhancing vaccine acceptance in such communities [114,115]. The 80% acceptance rate in Chinese communities may be indicative of the influence of community norms, government policies, and public health campaigns in shaping vaccine perceptions. Understanding the sociocultural dynamics and leveraging community influences can be instrumental in developing effective strategies to enhance vaccine acceptance in different cultural contexts.

This umbrella review, although offering insights into global vaccine acceptance and hesitancy, does possess limitations inherent to the studies included. The substantial heterogeneity across these studies hinders the ability to draw definitive conclusions and underscores the necessity for a cautious interpretation of the findings. Variations in study designs, targeted populations, time frames, and geographic locations highlight the need for standardization in future research to improve comparability and generalizability. Our review only included articles published in English. The overlap of the same primary studies is inevitable; different systematic reviews might have included the same primary studies.

Future research should focus on exploring the underlying factors influencing vaccine acceptance and hesitancy in diverse populations and contexts. Qualitative studies can provide in-depth insights into individual beliefs, perceptions, and information needs, enabling the development of targeted interventions. Longitudinal studies can assess the temporal variations in vaccine perceptions and the impact of evolving information landscapes on vaccine-related behaviors.

## Appendix

Multimedia Appendix 1 PRISMA Checklist.

Multimedia Appendix 2 Supplementary tables.

Multimedia Appendix 3 Forest plot illustrating the pooled vaccine acceptance rate for individuals with chronic diseases.

Multimedia Appendix 4 Forest plot depicting the vaccine acceptance rate among health care workers.

Multimedia Appendix 5 Forest plot depicting the vaccine acceptance rate among pregnant women.

Multimedia Appendix 6 Pooled vaccine acceptance rate of parents consenting for their children.

Multimedia Appendix 7 Vaccine acceptance rates for Chinese community residents, migrants, and refugees.

## Abbreviations

AMSTAR: A Measurement Tool to Assess Systematic Reviews
PRISMA: Preferred Reporting Items for Systematic Reviews and Meta-Analyses
WHO: World Health Organization
