# Corrigendum to: COVID-19 Vaccine Hesitancy: Umbrella Review of Systematic Reviews and Meta-Analysis

**DOI:** 10.2196/64080

**Published:** 2024-07-10

**Authors:** Tiffany Leung, Travis Sanchez, Gunther Eysenbach

**Affiliations:** 1 JMIR Publications Toronto, ON Canada; 2 Department of Epidemiology, Rollins School of Public Health, Emory University Atlanta, GA United States

Shortly after the publication of the article titled “COVID-19 Vaccine Hesitancy: Umbrella Review of Systematic Reviews and Meta-Analysis” in *JMIR Public Health and Surveillance* [1], the editors discovered processing errors that occurred during the peer review process. Specifically, Roy Rillera Marzo, MD, acted as guest editor for the theme issue titled “Preventive Strategies: Scaling Up Effective Public Health Interventions for Long-Term Population Health Benefits” and made a decision for this paper where they are also a coauthor, which is against the Committee on Publication Ethics (COPE) [2] and JMIR Publications’ Conflict of Interest policies [3].

As a result, the paper underwent additional post-publication editorial review to address the question whether the published version should be retracted or can stand. During the post-publication review process, the editor in chief of *JMIR Public Health and Surveillance,* Travis Sanchez, DVM, MPH, reviewed the manuscript drafts, peer review comments, and author responses. He noted that the paper adheres to standards for systematic reviews. There were two reviews on record, including one peer review that identified methodological issues that the authors addressed in a subsequent revision.

After the first round of reviews, guest editor Marzo did not adhere to JMIR Publications’ best practices and decision-making guidelines for editors by issuing a “B” decision (ie, minor revisions, no further peer review required) instead of a “C” decision (ie, revise and re-review), which would have presented the revised manuscript to the peer reviewers for re-review [4]. Upon editorial review of the revised manuscript and the authors’ point-by-point responses to the peer reviewers’ comments, it was noted that there could have been additional clarifications in the author responses, including more explicit comments on impacts of the changes made on the results. However, on examination of the edits that the authors made in the final draft of the manuscript by the editor in chief, it appears that the authors appropriately addressed the most substantive methodology concerns noted by the peer reviewers. Although minor revisions or refinements could have been made, the validity of the study appears to be sound and would not be impacted by minor revisions. No further revisions, corrections, or addenda are required at this time.

As a result of our investigations and a de novo evaluation of the acceptability of the paper by the editor in chief Travis Sanchez, the determination is that the editor of record for this manuscript has been corrected to Travis Sanchez. The editor information in the published paper will be changed from:

Edited by R Marzo

to:

Edited by T Sanchez

At the time of this manuscript publication, Marzo had not made other decisions for this theme issue. Henceforward, Marzo and other guest editors will only make future decisions for this theme issue together with editor in chief Sanchez.

JMIR Publications adheres to COPE guidance, including the 2023 published guidance on “Best practices for guest edited collections” [2]. JMIR Publications also routinely reviews and updates its Conflict of Interest disclosure policies as part of a larger effort to promote integrity of the published scientific record [3]. JMIR Publications has adhered to all key points from the COPE guidance on guest edited collections, and processes have been further optimized to mitigate risks and prevent further similar occurrences.

The correction of the editor’s name will appear in the online version of the paper on the JMIR Publications website on July 10, 2024, together with the publication of this correction notice. Because this correction was made after submission to PubMed, PubMed Central, and other full-text repositories, the corrected article has also been resubmitted to those repositories.
