# Supplementary material for: COVID-19 Vaccine Hesitancy: Umbrella Review of Systematic Reviews and Meta-Analysis
Source: JMIR Public Health Surveill. 2024 Apr 30;10:e54769. doi: 10.2196/54769 (PMC11062401; doi:10.2196/54769)
Supplement: Multimedia Appendix 2 [file publichealth_v10i1e54769_app2.pdf]

Table S1. Inclusion and Exclusion criteria

Research Question: "Covid-19 vaccine acceptance and hesitance rate"

	Inclusion	Exclusion
**Participants**	General population<br>Specific subgroups of population	None
**Intervention**	None	
**Outcome**	Rate of acceptance and hesitance for COVID-19 vaccine	Other vaccines
**Study Designs**	Systematic reviews and meta-analysis of Observational studies	Clinical trials,<br>Letter to editor,<br>Commentaries,<br>Abstract only, Case series, case reports, reviews, Discussion papers, animal studies, and systematic reviews without meta-analysis
	Date of Search- 15th of September 2023<br>Published articles and preprints data with no language restriction	Unavailable full-text articles

Table S2. The adjusted search terms as per searched electronic databases [as of 15.09.2023]

Database	No	Search Query	Results
**PubMed**			
	#1	(("Acceptance"[Title/Abstract] OR "Willingness"[Title/Abstract] OR "Unwillingness"[Title/Abstract] OR "Hesitancy"[Title/Abstract] OR "Intention"[Title/Abstract] OR "Unacceptance"[Title/Abstract]) AND ("vaccin*"[Title] OR "Immunization"[Title]) AND ("meta analysis"[Title/Abstract] OR "Metanalysis"[Title/Abstract] OR "meta analysis"[Title/Abstract] OR "Systematic review"[Title/Abstract]) AND ("corona*"[Title] OR "covid*"[Title] OR "SARS-CoV-2"[Title])) OR (("Acceptance"[Title/Abstract] OR "Willingness"[Title/Abstract] OR "Unwillingness"[Title/Abstract] OR "Hesitancy"[Title/Abstract] OR "Intention"[Title/Abstract] OR "Unacceptance"[Title/Abstract]) AND ("vaccin*"[Title] OR "Immunization"[Title]) AND ("corona*"[Title] OR "covid*"[Title] OR "SARS-CoV-2"[Title]) AND ("meta analysis"[Publication Type] OR "Systematic review"[Filter]))	199
**Scopus**			
	#1	(TITLE ("corona*" OR "covid*" OR "SARS-CoV-2") AND TITLE ("vaccin*" OR "immunization") AND TITLE ("Meta-analysis" OR "systematic review") AND TITLE-ABS-KEY (acceptance OR willingness OR unwillingness OR hesitancy OR hesitance OR intention OR unacceptance))	164
**Embase**			
	#1	('coronavirus disease 2019'/exp OR 'coronavirus disease 2019') AND ('meta-analysis':ti OR 'systematic review':ti) AND ('vaccin*':ti OR 'immunization':ti) AND (acceptance:ab,ti OR willingness:ab,ti OR unwillingness:ab,ti OR hesitancy:ab,ti OR hesitance:ab,ti OR intention:ab,ti OR unacceptance:ab,ti)	137
**Web of Science**			
	#1	(TS=("corona*" OR "covid*" OR "SARS-CoV-2") AND TS=("vaccin*" OR "immunization")) AND (TS=("meta-analysis" OR "metaanalysis" OR "metanalysis" OR "systematic review")) AND (TS=("acceptance" OR	221

	"willingness" OR "unwillingness" OR "hesitancy" OR "hesitance" OR "intention" OR "unacceptance"))	
**Cochrane**		
#1	Title/Abstract ("corona*" OR "covid*" OR "SARS-CoV-2") AND Title/Abstract ("vaccin*" OR "immunization") AND Title/Abstract ("Meta-analysis") AND Title/Abstract (acceptance OR willingness OR unwillingness OR hesitancy OR hesitance OR intention OR unacceptance))	4

Table S3. Overview of Included Meta-Analyses (2021-2023)

Author, Year	No. of studies	No. of participants	Date of search	Populations	Outcomes evaluated	Small study effects and P value	Overall quality (Amstar2)
Abdelmoneim et al, 2022 [23]	48	NA	04-06-22	General population	Acceptance	NA	Moderate
Abu El Kheir-Mataria et al, 2023 [86]	13	NA	02-2022	Parents for their children	Acceptance	NA	Moderate
Ackah et al, 2022 [53]	21	14132	09-21	Healthcare workers	Acceptance	*P*= .16	Moderate
Akem Dimala et al, 2021 [41]	32	70997	31-11-22	General population	Acceptance	NA	Moderate
Alarcón-Braga et al, 2022 [27]	18	518 941	20-09-21	General population	Acceptance	NA	low
Alemayehu et al, 2022 [31]	25	33044	28-02-22	General population	Acceptance	*P*= .003	Moderate
Alimohamadi et al, 2022 [10]	54	NA	NA	General population, Healthcare workers	Acceptance	NA	Critically low
Alimoradi et al, 2023 [70]	19	14,943	07-22	Migrant and refugee groups	Acceptance	*P*=.15	High
Alimoradi et al, 2023 [69]	98	413590	07-22	Parents for their children	Acceptance	*P*= < .001	Moderate
Azami et al, 2022 [64]	16	19219	10-2021	Pregnant women	Acceptance	*P* = .88	High
Azanaw et al, 2022 [36]	29	26255	NA	General population	Acceptance	*P* = .003	High
Belay et al, 2022 [33]	14	6,773	12-10-21	General population	Acceptance	*P*= .07	Moderate
Bhattacharya et al, 2022 [63]	17	25 147	01-04-22	Pregnant women	Acceptance	*P*= < .05	Moderate

Bianchi et al, 2023 [84]	7	NA	21-03-22	Persons with diabetes	Hesitance	*P*=.85	Moderate
Bianchi et al, 2022 [80]	46	NA	06-02-22	Pregnant and breastfeeding women	Hesitance	NA	Moderate
Bianchi et al, 2022 [78]	14	NA	25-01-22	Health care workers	Hesitance	NA	Moderate
Bianchi et al, 2023 [81]	9	NA	23-07-22	Parents for children	Hesitance	NA	Moderate
Cénat et al, 2022 [75]	24	136 889	01-03-22	General population	Hesitance, Unwillingness	$z = -1.71$, $P = .08$	low
Chen et al, 2022 [67]	29	68327	06-11-21	Parents for their children	Acceptance	NA	High
Ejamo et al, 2023 [49]	14	NA	01-09-21	People living with HIV	Acceptance	*P* = .47	Moderate
Ekpor and Akyirem, 2023 [50]	18	11292	20-11-22	Persons with diabetes	Acceptance	Peters' test with *P*= .34	High
Fajar et al, 2022 [74]	56	NA	25-05-22	General population	Hesitance	*P*= 1.27	High
Galanis et al, 2022 [66]	43, 34	NA	12-12-21	Parents for their children, Parents for their children	Willingness, Hesitance	*P* < .05	High
Galanis et al, 2022 [62]	11	703004	23-03-2022	Pregnant women	Acceptance	*P* <.05	low
Geng et al, 2022 [87]	34	42543	31-12-21	College students	Acceptance	*P* = .41	Moderate
Gudayu and Mengistie, 2023 [30]	35	38460	05-06-22	General population	Acceptance	NA	Moderate
Hajissa et al, 2023 [71]	14	29152	NA	Migrants, Refugees, and Foreign Workers	Acceptance, Hesitance	NA	low
Halemani et al, 2022 [60]	24	26,995	10-03-22	Pregnant women	Acceptance	NA	critically low
Islam et al, 2023 [17]	43	60,852	31-12-21	General population	Hesitance	*P*< .001	critically low
Kawuki et al, 2023 [32]	24	30323	19-01-23	General population	Acceptance, Hesitance	*P* = .50	critically low

Kazeminia et al, 2022 [43]	98	660,604	05-2021	General population	Acceptance	*P*< .05	Moderate
Khabour, 2022 [24]	22	38600	20-04-22	General population	Acceptance	NA	Moderate
Kigongo et al, 2023 [82]	15	7498	10-22	health care workers	Hesitance	*P*=.32	High
Kukreti et al, 2022 [39]	19	33844	01-04-21	General population	Acceptance	*P*=.67	Moderate
Kumar et al, 2023 [16]	23	39567	09-09-22	General population	Acceptance	*P*= .49	Moderate
Lin et al, 2022 [85]	10	NA	10-2021	Among dental students	Acceptance	*P*= .11	High
Lin et al, 2022 [47]	10	2589	30-04-22	patients with epilepsy and their caregivers	Acceptance	NA	Moderate
Liu and Kuang, 2023 [90]	161	705957	12-05-22	General population	Acceptance	NA	low
Luo et al, 2021 [11]	9	24952	19-02-21	Health care workers	Willingness	NA	Moderate
Ma et al, 2022 [68]	13	47994	01-06-22	Parents for their children	Acceptance	*P* > .05	High
Mahmud et al, 2022 [35]	79	1,581,562	25-04-21	General population and Healthcare workers	Acceptance	Z = -2.95, *P* = .003	High
Mekonnen and Mengistu, 2022 [28]	14	6373	10-10-21.	General population	Acceptance	*P* = .82	High
Mengistu et al, 2022 [29]	68	143111	26-06-22	General population	Acceptance	NA	low
Meybodi et al, 2022 [48]	24	NA	11-11-21	Patients with inflammatory bowel disease	Acceptance	NA	low
Moltot et al, 2023 [54]	11	5971	20-08-22	Health care professionals	Acceptance	*P*= .38	low
Mose et al, 2022 [44]	12	5029	07-01-22	General population	Acceptance	*P* >.05	Moderate
Nassr et al, 2022 [59]	12	16926	05-2021.	Pregnant women	Acceptance	NA	Moderate
Nehal et al, 2021 [15]	63	NA	01-03-21	General population	Acceptance	NA	low
Nikpour et al, 2022 [58]	10	16, 696	11-07-21	Pregnant women	Acceptance	NA	Moderate

Nindrea et al, 2022 [88]	12	NA	01-12-21	Pregnant women	Acceptance	NA	Moderate
Nindrea et al., 2021 [89]	24	NA	03-21	General population	Acceptance	NA	critically low
Nnaemeka et al, 2023 [40]	42	24,533	05-22	General population	Acceptance, Hesitance	*P* =.40	Moderate
Norhayati et al, 2022 [12]	170	814691	01-07-21	General population	Acceptance	NA	Moderate
Patwary et al, 2022 [79]	31	30272	05-03-22	Healthcare Students	Acceptance, Hesitance	*P* =.64	High
Patwary et al, 2022 [73]	36	83867	15-01-22	General population among Low- and Lower-Middle-Income Countries	Acceptance, Hesitance	*P* =.02	Moderate
Politis et al, 2023 [56]	22	24,882	01-11-22	health care workers	Acceptance	*P* = .86	Moderate
Prabani et al, 2022 [51]	20	NA	06-09-21	Patients with cancer	Acceptance	NA	low
Renzi et al, 2022 [42]	71	NA	04-21	General population	Acceptance	NA	low
Ripon et al, 2022 [77]	20	2962/7384	01-09-22	Black/African American	Hesitance	*P* = .654	High
Robinson et al, 2021 [34]	28	58656	01-11-20	General population	Acceptance	z = 3.69, *P* <.001	High
Sahile et al, 2022 [25]	18	10873	NA	General population	Acceptance	NA	Moderate
Sarantaki et al, 2022 [57]	18	NA	03-10-20	Pregnant women	Acceptance	NA	critically low
Shamshirsaz et al, 2022 [61]	12	NA	22-05-22	Pregnant women	Acceptance	NA	low
Shui et al, 2022 [52]	18	45,760	01-06-22	Health care workers	Acceptance	Egger's regression test (t = -0.74, *P* = .47)	low
Terry et al, 2022 [37]	18	5447	12-02-21	General population	Acceptance	NA	Moderate

Ulbrichtova et al, 2022 [55]	6	4118	12-21	Medical Students	Acceptance	NA	Moderate
Veronese et al, 2021 [76]	15	9753	18-06-21	Older adults	Unwillingness, Uncertainty	Egger's test = 1.77 ± 8.59 ; *P* = .84	High
Wake 2021 [26]	22	33912	14-06-21	General population	Acceptance	*P* =.23	High
Wang et al, 2021 [22]	28, 7, 1	81173	04-11-20	General population, Health care workers, Chronic disease	Acceptance, Acceptance, Acceptance	NA	Moderate
Wang et al, 2022 [83]	519	7990117	27-02-22	General population	Acceptance	NA	low
Wang et al, 2022 [13]	71	93508	15-05-22	Health care workers	Acceptance	*P* = .049	High
Wang et al, 2022 [65]	12	22902	14-07-05	Parents for their children	Acceptance	*P* > .05	Moderate
Worede et al, 2023 [14]	8	4419	30-01-23	Pregnant women	Acceptance	*P*=.65	Moderate
Xu and Zhu, 2022 [72]	12	29278	25-08-22	Chinese community residents	Acceptance	*P* > .05	Moderate
Yanto et al, 2022 [9]	109	1166275	25-08-22	General population	Acceptance	Begg and Mazumdar's test :- *P*<.001, Egger's = *P* <0.001	High
Yazdani et al, 2022 [45]	10	5983	01-10-21	Patients with multiple sclerosis	Willingness	NA	Moderate
Yenew et al, 2023 [38]	19	47873	14-10-21	General population	Acceptance	*P* =.163	Moderate
Zhao et al, 2023 [46]	31	57875	01-10-22	People with chronic disease	Acceptance	NA	low

Table S4. Summary of quality assessment of included systematic reviews using AMSTAR2

Study	1	2	3	4	5	6	7	8	9	10	11	12	13	14	15	16	
	Non-critical domain	Critical domain	Non-critical domain	Critical domain	Non-critical domain	Non-critical domain	Critical domain	Non-critical domain	Critical domain	Non-critical domain	Critical domain	Non-critical domain	Critical domain	Non-critical domain	Critical domain	Non-critical domain	Overall
Abdelmoneim et al [23]	Yes	Yes	Yes	Yes	No	No	Yes	Yes	Yes	NO	Yes	No	No	No	NO	Yes	Moderate
Abu El Kheir-Mataria et al [86]	Yes	Yes	Yes	Yes	No	No	Yes	Yes	Yes	NO	Yes	No	No	No	NO	Yes	Moderate
Ackah et al [53]	Yes	Yes	Yes	Yes	No	No	Yes	Yes	Yes	NO	Yes	No	No	No	Yes	Yes	Moderate
Akem Dimala et al [41]	Yes	Yes	Yes	Yes	No	No	Yes	Yes	Yes	NO	Yes	Yes	Yes	no	NO	Yes	Moderate
Alarcón-Braga et al [27]	Yes	Yes	Yes	Yes	No	No	Yes	Yes	Yes	NO	Yes	No	No	No	NO	Yes	low
Alemayehu et al [31]	Yes	Yes	Yes	Yes	No	No	Yes	Yes	Yes	NO	Yes	No	No	No	Yes	Yes	Moderate
Alimohamadi et al [10]	No	Yes	Yes	Yes	No	No	Yes	Yes	no	NO	Yes	No	No	No	NO	Yes	critically low
Alimoradi et al [70]	Yes	Yes	Yes	Yes	No	No	no	Yes	Yes	NO	Yes	No	No	No	Yes	Yes	High
Alimoradi et al [69]	Yes	Yes	Yes	Yes	No	No	Yes	Yes	Yes	NO	Yes	yes	No	No	Yes	Yes	Moderate
Azami et al [64]	Yes	Yes	Yes	Yes	No	No	Yes	Yes	Yes	NO	Yes	No	No	No	Yes	Yes	High
Azanaw et al [36]	Yes	Yes	Yes	Yes	No	No	Yes	Yes	Yes	NO	no	yes	Yes	no	Yes	Yes	High
Belay et al [33]	Yes	Yes	Yes	Yes	No	No	Yes	no	Yes	NO	Yes	yes	No	No	Yes	Yes	Moderate
Bhattacharya et al [63]	Yes	Yes	Yes	Yes	No	No	Yes	Yes	Yes	NO	Yes	No	No	No	Yes	Yes	Moderate
Bianchi et al [84]	Yes	Yes	Yes	Yes	No	No	Yes	Yes	Yes	NO	Yes	No	No	No	Yes	Yes	Moderate
Bianchi et al [80]	Yes	Yes	Yes	Yes	No	No	no	Yes	Yes	NO	Yes	No	No	No	no	Yes	Moderate
Bianchi et al [78]	Yes	Yes	Yes	Yes	No	No	Yes	Yes	no	NO	Yes	No	No	No	no	Yes	Moderate
Bianchi et al [81]	Yes	Yes	Yes	Yes	No	No	Yes	Yes	Yes	NO	no	yes	Yes	no	NO	Yes	Moderate
Cénat et al [75]	Yes	Yes	Yes	Yes	No	No	Yes	Yes	Yes	NO	Yes	no	no	no	Yes	Yes	low
Chen et al [67]	Yes	Yes	Yes	Yes	No	No	Yes	Yes	Yes	NO	Yes	No	No	No	Yes	Yes	High
Ejamo et al [49]	Yes	Yes	Yes	Yes	No	No	Yes	no	Yes	NO	Yes	No	No	No	Yes	Yes	Moderate
Ekpor and Akyirem [50]	Yes	Yes	Yes	Yes	No	No	Yes	Yes	Yes	NO	Yes	yes	Yes	no	Yes	Yes	High
Fajar et al [74]	Yes	Yes	Yes	Yes	No	No	no	Yes	Yes	NO	Yes	yes	yes	No	Yes	Yes	High
Galanis et al [66]	Yes	Yes	Yes	Yes	No	No	Yes	Yes	Yes	NO	Yes	No	No	No	Yes	Yes	High
Galanis et al [62]	Yes	Yes	Yes	Yes	No	No	Yes	Yes	Yes	NO	no	No	No	No	Yes	Yes	low
Geng et al [87]	Yes	Yes	Yes	Yes	No	No	Yes	Yes	no	NO	Yes	No	No	No	Yes	Yes	Moderate
Gudayu and Mengistie [30]	Yes	Yes	Yes	Yes	No	No	Yes	Yes	Yes	NO	Yes	No	No	No	NO	Yes	Moderate
Hajissa et al [71]	Yes	Yes	Yes	Yes	No	No	Yes	Yes	Yes	NO	Yes	No	No	No	NO	Yes	low
Halemani et al [60]	Yes	Yes	Yes	Yes	No	No	Yes	Yes	Yes	NO	Yes	No	No	No	NO	Yes	critically low
Islam et al [17]	Yes	Yes	Yes	Yes	No	No	Yes	Yes	Yes	NO	Yes	YEs	No	No	Yes	Yes	critically low
Kawuki et al [32]	Yes	no	Yes	Yes	No	No	Yes	Yes	Yes	NO	Yes	No	No	No	Yes	Yes	critically low
Kazeminia et al [43]	Yes	Yes	Yes	Yes	No	No	Yes	Yes	Yes	NO	Yes	No	No	No	Yes	Yes	Moderate
Khabour [24]	Yes	Yes	Yes	Yes	No	No	Yes	Yes	no	No	Yes	No	No	No	YEs	Yes	Moderate
Kigongo et al [82]	Yes	Yes	Yes	Yes	No	No	Yes	Yes	Yes	NO	Yes	No	No	No	Yes	Yes	High
Kukreti et al [39]	Yes	Yes	Yes	Yes	No	No	Yes	Yes	Yes	NO	no	No	No	No	Yes	Yes	Moderate

Kumar et al [16]	Yes	Yes	Yes	Yes	No	No	no	Yes	Yes	NO	Yes	No	No	No	Yes	Yes	Moderate
Lin et al [85]	Yes	Yes	Yes	Yes	No	No	Yes	Yes	Yes	NO	Yes	No	No	No	Yes	Yes	High
Lin et al [47]	Yes	Yes	Yes	Yes	No	No	no	Yes	Yes	NO	Yes	No	No	No	NO	Yes	Moderate
Liu and Kuang [90]	NO	Yes	Yes	Yes	No	No	Yes	Yes	Yes	NO	Yes	No	No	No	NO	Yes	low
Luo et al [11]	Yes	Yes	Yes	Yes	No	No	Yes	Yes	Yes	NO	Yes	No	No	No	Yes	Yes	Moderate
Ma et al [68]	Yes	Yes	Yes	Yes	No	No	Yes	Yes	Yes	NO	Yes	yes	No	No	Yes	Yes	High
Mahmud et al [35]	Yes	Yes	Yes	Yes	No	No	Yes	Yes	Yes	NO	Yes	No	No	No	Yes	Yes	High
Mekonnen and Mengistu [28]	Yes	Yes	Yes	Yes	No	No	Yes	Yes	Yes	NO	Yes	yes	yes	No	Yes	Yes	High
Mengistu et al [29]	Yes	Yes	Yes	Yes	No	No	Yes	no	Yes	NO	Yes	no	no	no	NO	Yes	low
Meybodi et al [48]	Yes	Yes	Yes	Yes	No	No	Yes	Yes	Yes	NO	Yes	No	No	No	NO	Yes	low
Moltot et al [54]	Yes	Yes	Yes	Yes	No	No	Yes	Yes	no	NO	Yes	No	No	No	Yes	Yes	low
Mose et al [44]	Yes	Yes	Yes	Yes	No	No	Yes	Yes	Yes	NO	Yes	No	No	No	Yes	Yes	Moderate
Nassr et al [59]	Yes	Yes	Yes	Yes	No	No	Yes	Yes	Yes	NO	Yes	No	No	No	NO	Yes	Moderate
Nehal et al [15]	Yes	Yes	Yes	Yes	No	No	Yes	Yes	Yes	NO	no	No	No	No	Yes	Yes	low
Nikpour et al [58]	Yes	Yes	Yes	Yes	No	No	Yes	Yes	Yes	NO	Yes	No	No	No	NO	Yes	Moderate
Nindrea et al [88]	Yes	Yes	Yes	Yes	No	No	Yes	Yes	Yes	NO	Yes	No	No	No	NO	Yes	Moderate
Nindrea et al [89]	NO	no	Yes	Yes	No	No	Yes	Yes	Yes	NO	Yes	No	No	No	NO	Yes	critically low
Nnaemeka et al [40]	Yes	Yes	Yes	Yes	No	No	Yes	Yes	Yes	NO	Yes	No	No	No	Yes	Yes	Moderate
Norhayati et al [12]	Yes	Yes	Yes	Yes	No	No	Yes	Yes	Yes	NO	Yes	No	No	No	NO	Yes	Moderate
Patwary et al [79]	Yes	Yes	Yes	Yes	No	No	Yes	Yes	Yes	NO	Yes	yes	yes	yes	Yes	Yes	High
Patwary et al [73]	Yes	Yes	Yes	Yes	No	No	no	Yes	Yes	NO	Yes	No	No	No	Yes	Yes	Moderate
Politis et al [56]	Yes	Yes	Yes	Yes	No	No	Yes	Yes	no	NO	Yes	No	No	No	Yes	Yes	Moderate
Prabani et al [51]	Yes	Yes	Yes	Yes	No	No	Yes	Yes	Yes	NO	Yes	No	No	No	NO	Yes	low
Renzi et al [42]	Yes	Yes	Yes	Yes	No	No	Yes	Yes	Yes	NO	Yes	No	No	No	No	Yes	low
Ripon et al [77]	Yes	Yes	Yes	Yes	No	No	Yes	Yes	Yes	NO	Yes	no	no	no	Yes	Yes	High
Robinson et al [34]	Yes	Yes	Yes	Yes	No	No	Yes	Yes	Yes	NO	yes	yes	Yes	no	Yes	Yes	High
Sahile et al [25]	Yes	Yes	Yes	Yes	No	No	Yes	Yes	Yes	NO	no	No	No	No	NO	Yes	Moderate
Sarantaki et al [57]	Yes	Yes	Yes	Yes	No	No	Yes	Yes	Yes	NO	Yes	No	No	No	NO	Yes	critically low
Shamshirsaz et al [61]	Yes	Yes	Yes	Yes	No	No	Yes	Yes	Yes	NO	Yes	No	No	No	no	Yes	low
Shui et al [52]	Yes	Yes	Yes	Yes	No	No	No	Yes	Yes	NO	Yes	No	No	No	Yes	Yes	low
Terry et al [37]	Yes	Yes	Yes	Yes	No	No	Yes	Yes	no	NO	Yes	No	No	No	NO	Yes	Moderate
Ulbrichtova et al [55]	Yes	Yes	Yes	Yes	No	No	Yes	Yes	Yes	NO	Yes	No	No	No	NO	Yes	Moderate
Veronese et al [76]	Yes	Yes	Yes	Yes	No	No	Yes	Yes	Yes	NO	Yes	yes	yes	No	Yes	Yes	High
Wake [26]	Yes	Yes	Yes	Yes	No	No	Yes	Yes	Yes	NO	Yes	yes	yes	yes	Yes	Yes	High
Wang et al [22]	Yes	Yes	Yes	Yes	No	No	Yes	Yes	Yes	No	Yes	No	Yes	Yes	Yes	Yes	Moderate
Wang et al [83]	Yes	Yes	Yes	Yes	No	No	Yes	Yes	Yes	NO	Yes	yes	No	No	NO	Yes	low
Wang et al [13]	Yes	Yes	Yes	Yes	No	No	Yes	Yes	Yes	NO	Yes	yes	Yes	no	Yes	Yes	High
Wang et al [65]	Yes	Yes	Yes	Yes	No	No	Yes	Yes	Yes	NO	Yes	No	No	No	Yes	Yes	Moderate
Worede et al [14]	Yes	Yes	Yes	Yes	No	No	Yes	Yes	Yes	NO	Yes	No	No	No	Yes	Yes	Moderate
Xu and Zhu [72]	Yes	Yes	Yes	Yes	No	No	Yes	Yes	no	NO	Yes	yes	yes	No	Yes	Yes	Moderate
Yanto et al [9]	Yes	Yes	Yes	Yes	No	No	Yes	Yes	Yes	NO	Yes	No	No	No	Yes	Yes	High

Yazdani et al [45]	Yes	Yes	yes	Yes	No	No	Yes	Yes	Yes	No	Yes	No	No	No	NO	Yes	Moderate
Yenew et al [38]	Yes	Yes	Yes	Yes	No	No	Yes	Yes	Yes	NO	Yes	No	No	No	Yes	Yes	Moderate
Zhao et al [46]	Yes	Yes	Yes	Yes	No	No	Yes	Yes	Yes	NO	Yes	No	No	No	no	Yes	low
