# Supplementary figures and images for: COVID-19 Vaccine Hesitancy: Umbrella Review of Systematic Reviews and Meta-Analysis
Source: JMIR Public Health Surveill. 2024 Apr 30;10:e54769. doi: 10.2196/54769 (PMC11062401; doi:10.2196/54769)

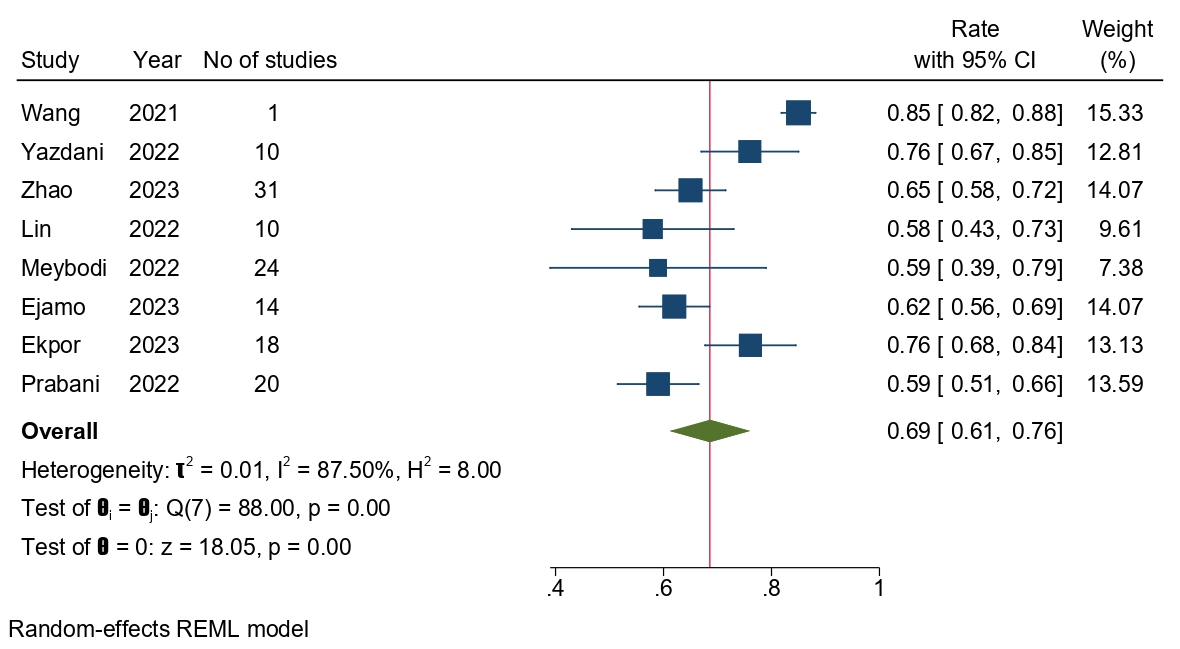

Supplement: Multimedia Appendix 3 [file publichealth_v10i1e54769_app3.png]

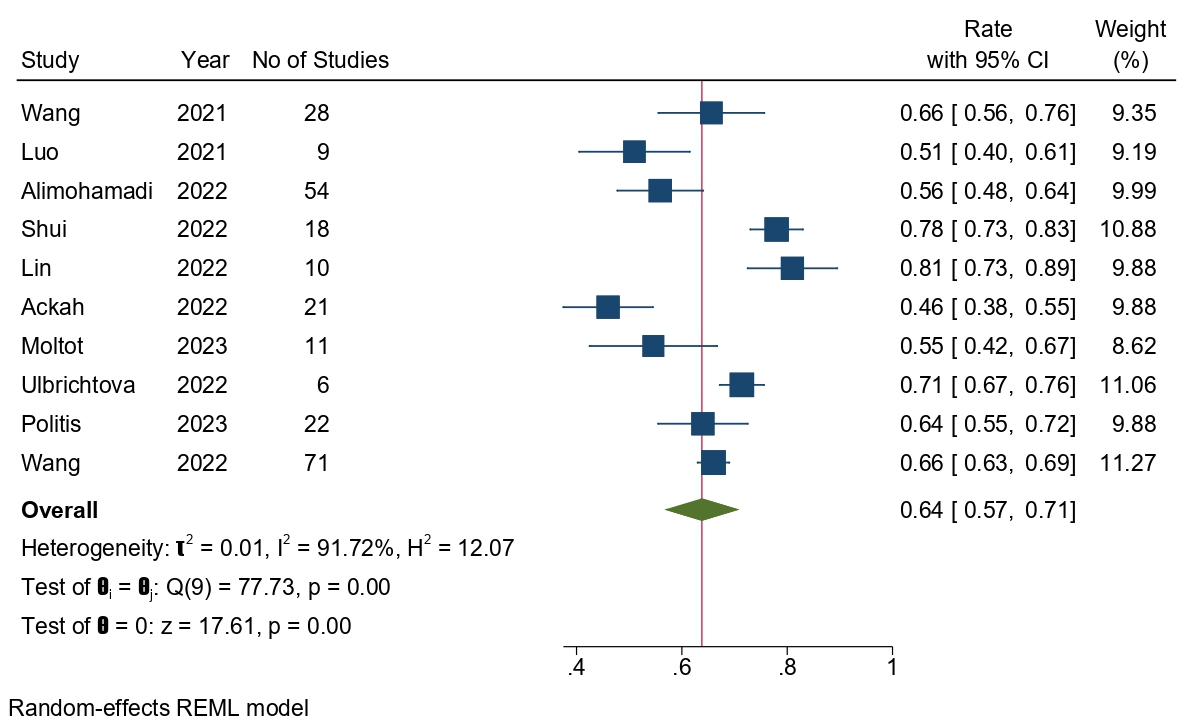

Supplement: Multimedia Appendix 4 [file publichealth_v10i1e54769_app4.png]

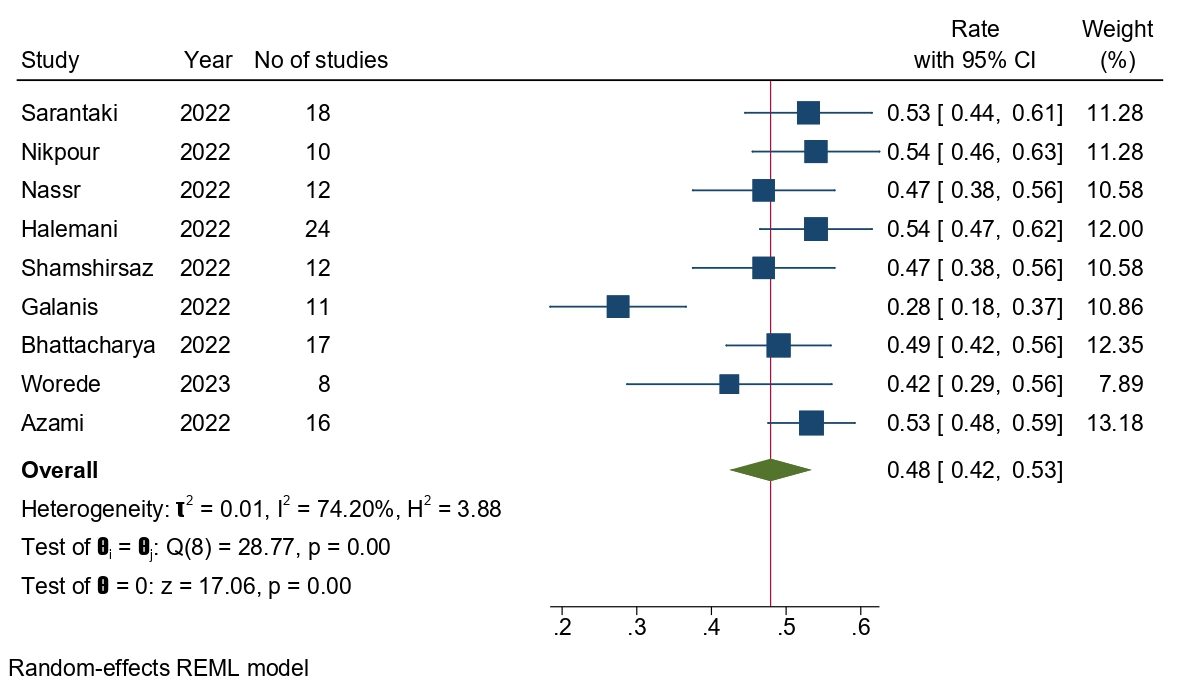

Supplement: Multimedia Appendix 5 [file publichealth_v10i1e54769_app5.png]

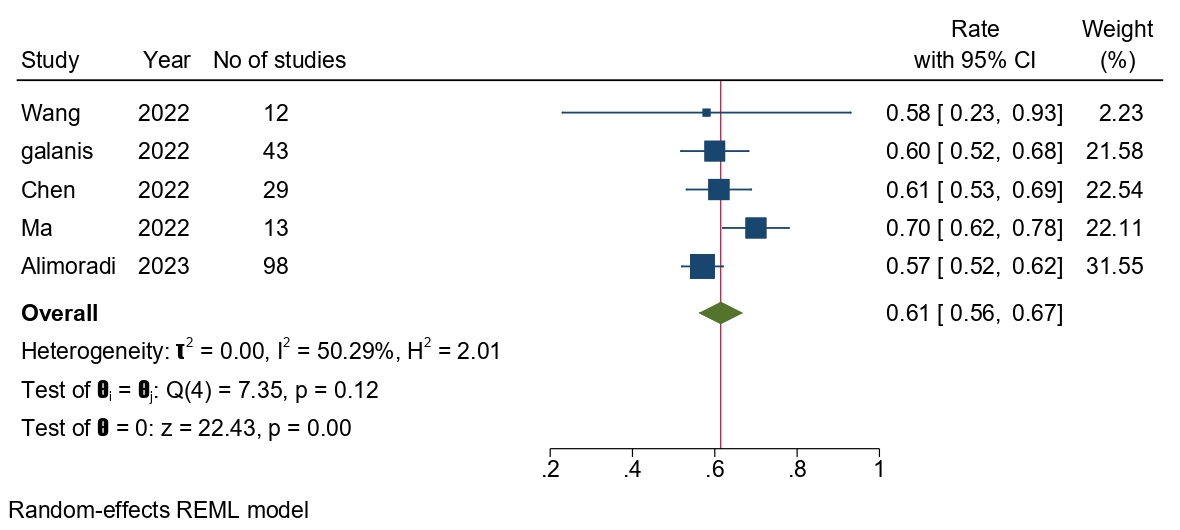

Supplement: Multimedia Appendix 6 [file publichealth_v10i1e54769_app6.png]

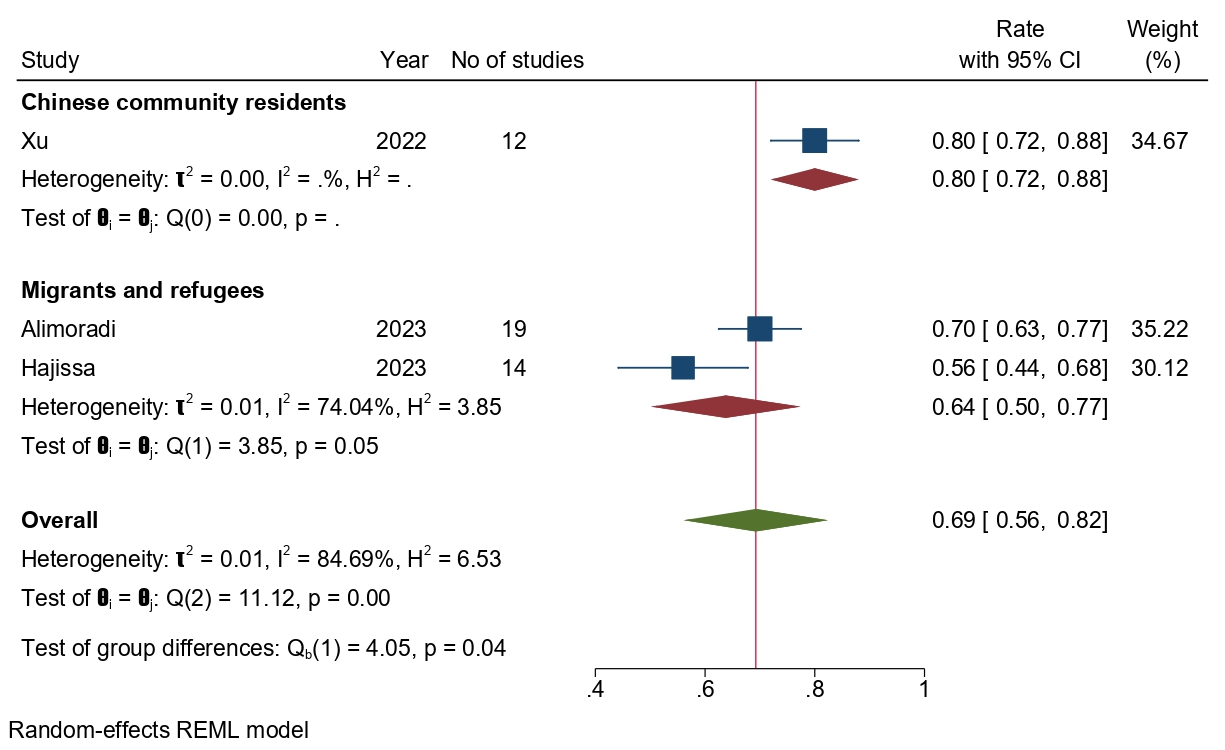

Supplement: Multimedia Appendix 7 [file publichealth_v10i1e54769_app7.png]
